# Performance Degradation of Ga_2_O_3_-Based X-Ray Detector Under Gamma-Ray Irradiation

**DOI:** 10.3390/mi16030339

**Published:** 2025-03-14

**Authors:** Xiao Ouyang, Silong Zhang, Tao Bai, Zhuo Chen, Yuxin Deng, Leidang Zhou, Xiaojing Song, Hao Chen, Yuru Lai, Xing Lu, Liang Chen, Liangliang Miao, Xiaoping Ouyang

**Affiliations:** 1 School of Physics and Astronomy, Beijing Normal University, Beijing 100875, China; oyx16@tsinghua.org.cn; 2 School of Materials Science and Engineering, Xiangtan University, Xiangtan 411105, China; 3 Northwest Institute of Nuclear Technology, Xi’an 710024, China; 4 School of Microelectronics, Xi’an Jiaotong University, Xi’an 710049, China; 5 State Key Laboratory of Optoelectronic Materials and Technologies, School of Electronics and Information Technology, Sun Yat-sen University, Guangzhou 510275, China; 6 Xi’an Engineering Research Center of Advanced 3D Vision, Biyuan 3rd Road, Xi’an 710000, China

**Keywords:** gallium oxide, hetero-junction, semiconductor detector, X-ray detection, γ radiation

## Abstract

X-ray response performances of a p-NiO/*β*-Ga_2_O_3_ hetero-junction diode (HJD) X-ray detector were studied before and after γ-ray irradiation at −200 V, with a total dose of 13.5 kGy(Si). The response performances of the HJD X-ray detector were influenced by the trap-assistant conductive process of the HJD under reverse bias, which exhibited an increasing net (response) current, nonlinearity, and a long response time. After irradiation, the Poole–Frenkel emission (PFE) dominated the leakage current of HJDs due to the higher electric field caused by the increased net carrier concentration of *β*-Ga_2_O_3_. This conductive process weakened the performance of the HJD X-ray detector in terms of sensitivity, output linearity, and response speed. This study provided valuable insights into the radiation damage and performance degradation mechanisms of Ga_2_O_3_-based radiation detectors and offered guidance on improving the reliability and stability of these radiation detectors.

## 1. Introduction

The ultra-wide bandgap semiconductor gallium oxide (Ga_2_O_3_) has the characteristics of low intrinsic carrier concentration and high irradiation resistance, which make Ga_2_O_3_-based devices promising prospects in the applications of high-energy physics, nuclear reaction monitoring, and space electronics [1,2,3,4,5]. Nowadays, Ga_2_O_3_-based radiation detectors have drawn great attention and have been designed for neutrons, charged particles, and X-ray detection [6,7,8,9,10]. However, when these radiation detectors are exposed to radiation during operation, their performance degrades. Although the effects of radiation on Ga_2_O_3_-based devices have been widely investigated recently [11,12,13,14,15], their impact on the performance of radiation detectors has rarely been studied. Gamma rays (γ-rays) are a type of high-energy electromagnetic radiation produced by the de-excitation of atomic nuclei. Their prevalent presence in the universe and nuclear fusion environments, coupled with the challenges associated with effective shielding, has led to significant interest in studying the total dose radiation damage inflicted by γ-rays on semiconductor devices [16,17,18]. Therefore, studying the degradation of detector performance in a γ radiation environment helps explore the service life of detectors in space and radiation monitoring applications.

In this paper, a p-NiO/*β*-Ga_2_O_3_ hetero-junction diode (HJD) X-ray detector was fabricated and tested under X-ray irradiation before and after gamma ray exposure. The gamma radiation was provided by a ^60^Co source, which provided a dose rate of 0.5 Gy(Si)∙s^−1^ in the point of space where the detector was placed. The detector was irradiated at −200 V, the maximum value allowed by the Keysight B2902, which is also the operating bias voltage during X-ray detection. This online mode, which indicates that the detector was biased under irradiation, simulated an actual monitoring environment for the radiation detector. The current-voltage (*I*-*V*), capacitance-voltage (*C*-*V*), and temperature-dependent *I*-*V* (*I*-*V*-*T*) characteristics of the HJD X-ray detector were under dark conditions before and after irradiation. In addition, the X-ray response performances to X-ray dose rates and switching modes were conducted.

## 2. Experiments

**Figure 1:**
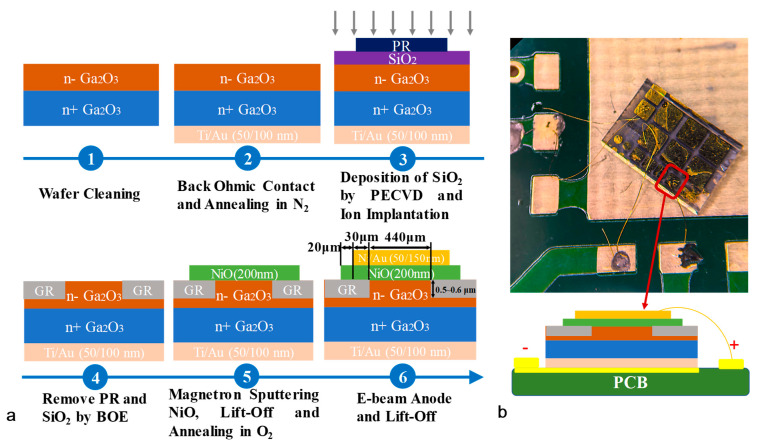
(**a**) The preparation process of NiO/*β*-Ga_2_O_3_ HJD X-ray detectors with N+-implanted incorporated guard ring. (**b**) The diagram of the fabricated detector packaged on a PCB.

A *β*-Ga_2_O_3_ sample from NCT company, with a lightly Si-doped epitaxial layer (grown by the hydride vapor phase epitaxy (HVPE) method) grown on a Sn-doped *β*-Ga_2_O_3_ (001)-oriented substrate (650 μm, with Sn doping concentration > 2 × 10^18^ cm^−3^), was used in this study. The p-NiO/*β*-Ga_2_O_3_ HJD X-ray detector was fabricated with an incorporated guard ring to reduce the high electric field at the edges of metal contacts. Figure 1a shows the preparation process of NiO/*β*-Ga_2_O_3_ HJDs. Initially, the samples were cleaned with acetone and isopropanol, followed by a four-cycle de-ionized (DI) water rinse and N_2_ blow drying. Subsequently, a Ti/Au metal layer (50 nm/100 nm) was deposited by electron beam evaporation as the cathode, followed by rapid thermal annealing at 470 °C for 60 s in N_2_ ambient to form ohmic contacts. A SiO_2_ layer was then deposited via plasma-enhanced chemical vapor deposition (PECVD) at 350 °C for 8 min as an implantation mask. The incorporated guard ring (GR) pattern was formed by photolithography, and triple-energy N⁺⁺ ion implantation was performed with doses of 8 × 10^12^ cm^−2^ (30 keV), 2.3 × 10^13^ cm^−2^ (160 keV), and 3 × 10^13^ cm^−2^ (360 keV), targeting a box-like doping profile (1.5 × 10^18^ cm^−3^, 0.5–0.6 μm in depth). The SiO_2_ mask and photoresist (PR) were subsequently removed by buffered oxide etch solution (BOE) etching. Then, a second photolithography step patterned the NiO region, followed by a 200 nm NiO film deposition via magnetron sputtering (5 mTorr, Ar/O_2_ = 1:1, 130 W) and lift-off process. Post-deposition annealing at 300 °C for 60 s in O_2_ ambient was applied to enhance NiO crystallinity. Finally, the anode structure, a 500 μm rounded-corner square (30 μm in radius), was defined by aligned photolithography, and a Ni/Au metal layer (50 nm/150 nm) was deposited via electron beam evaporation and lift-off to complete the device. After fabrication, the detector was packaged on a printed circuit board (PCB) to facilitate testing, as shown in Figure 1b.

The irradiation and radiation response experiments were conducted in a shielding room at room temperature. The HJD X-ray detector was designated for γ-ray irradiation with a total dose of 13.5 kGy(Si) at a bias voltage of −200 V. The γ-ray was generated by the ^60^Co source provided by the Northwest Institute of Nuclear Technology (NINT), and the dose rate at the irradiated HJD X-ray detector was 0.5 Gy(Si)∙s^−1^. Before and after the γ-ray irradiation, the typical current-voltage (*I*-*V*) and capacitance-voltage (*C*-*V*) characterizations in dark conditions and X-ray response performances of the HJD X-ray detector were measured by a Source Measure Unit (B2902A, Agilent). A hot plate (CH600-190) was used in the *I*-*V*-*T* measurement to provide the temperature for the detector. The X-ray source used in this study was a miniature X-ray tube with a tungsten anode (60 kV, 12W X-ray source, Moxtek, USA) provided by the NINT, which generates a wide continuous spectrum composed by the bremsstrahlung and the characteristic L-lines of tungsten. The average photon energy of X-ray photons was at around 10 keV when the X-ray tube accelerating voltage was set at 30 kV, and the X-ray dose rate was calibrated by Unidos dosimeter to be 383 mGy∙s^−1^ for air at a distance of 2 cm from the source. In addition, for the γ-ray and X-ray irradiation experiments, the detector was irradiated from the anode side. In this paper, all test results following irradiation refer specifically to on-line irradiation.

## 3. Results and Discussion

**Figure 2:**
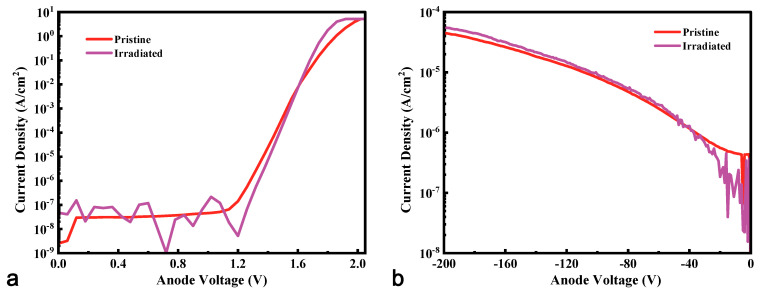
Typical (**a**) forward and (**b**) reverse *I*-*V* characteristics of pristine and irradiated HJDs X-ray detectors at 300 K.

Figure 2 displays the typical current-voltage (*I-V*) characteristics of pristine and irradiated HJDs at 300 K. The HJD X-ray detector exhibited similar *I*-*V* curves before and after γ-ray irradiation. Slight changes in the I-V curves of the HJD X-ray detector were observed after irradiation, such as higher forward current density, turn-on voltage, and leakage current density at high reversed bias. These results exhibited the opposite phenomenon against the study of the offline γ radiation effects (offline means the devices were irradiated without applied bias voltages) on the Ga_2_O_3_ diodes [19,20,21], which obtained higher on-resistance and lower leakage current after irradiation.

**Figure 3:**
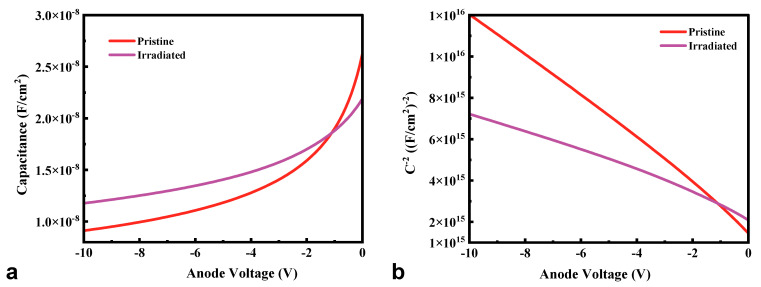
(**a**) Typical *C*-*V* and (**b**) *C*^−2^-*V* characteristics of the pristine and irradiated HJDs X-ray detectors at 300 K.

The *C*-*V* measurement was conducted to determine the reasons for the changes in the *I*-*V* curves shown in Figure 2. Figure 3a illustrates the typical *C-V* characteristics of the pristine and irradiated HJDs measured at 100 kHz. According to Equation (1), the net carrier concentration (*N*_D_-*N*_A_) can be extracted from the *C*^−2^-*V* curves shown in Figure 3b. Given the high doping concentration of ~10^19^ cm^−3^ for the NiO layer, the extracted net carrier concentration was mainly attributed to that of *β*-Ga_2_O_3_. Thus, the net carrier concentration of Ga_2_O_3_ increased from 1.38 × 10^16^ cm^−3^ to 3.07 × 10^16^ cm^−3^ after 13.5 kGy(Si) γ-ray irradiation, which was opposite to the decreased net carrier concentration revealed by offline radiation [19,20,21]. The difference between online and offline irradiation results was attributed to an extra electric field within the HJD detector when online irradiation occurred, and, in this case, the radiation-generated current exacerbated the impact of defects in the interface of NiO/Ga_2_O_3_ and the materials introduced by irradiation [22]. Consequently, the increased net carrier concentration of the *β*-Ga_2_O_3_ epitaxial layer caused by γ-ray irradiation reduced the on-resistance and increased the forward current. However, few papers have reported the increasing net carrier concentration after γ-ray irradiation, and the radiation damage mechanism in Ga_2_O_3_ should be studied further. In addition, the increased net carrier concentration in n-type Ga_2_O_3_ might have left up the fermi level of the electron and increased the barrier height between the p-NiO and n-Ga_2_O_3_, leading to a larger turn-on voltage after irradiation.

(1) $$N_{D}-N_{A}=-\frac{2}{q\varepsilon_{0}\varepsilon_{r}A^{2}}\frac{1}{\frac{dC^{-2}}{dV}}$$

where *C* is the measured capacitance of the HJD detector, *N_A_* is the acceptor concentration of *β*-Ga_2_O_3_, *N_D_* is the donor concentration of Ga_2_O_3_, *A* is the effective area of the HJD, *ε*_0_ is the permittivity of vacuum, and *ε_r_* is the static dielectric constant of the *β*-Ga_2_O_3_.

**Figure 4:**
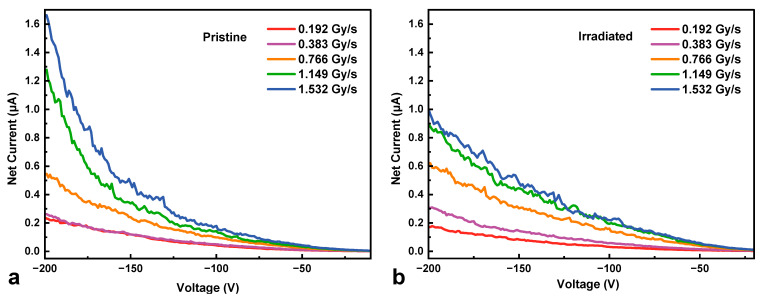
The net current of (**a**) pristine and (**b**) irradiated HJD X-ray detectors at various dose rates against bias voltages.

The X-ray response current was measured under the sweep mode from 0 V to −200 V, with a sampling rate of 1 V/60 ms. Figure 4 shows the net current (*I*_net_) of the HJD X-ray detector at various dose rates before and after irradiation, where the net current was defined as the output current under X-ray illumination (*I*_x-ray_) minus the dark current (*I*_dark_). The net current of the HJD X-ray detector increases with the bias voltage increasing both before and after irradiation. This phenomenon was observed in the Pt/Ga_2_O_3_ SBD X-ray detector [7] and the p-NiO/*β*-Ga_2_O_3_ HJD X-ray detector [23] without an incorporated guard ring, which was associated with leakage current due to the photoconductive mechanism caused by traps/defects in the Ga_2_O_3_ material. Moreover, because the net carrier concentration increased after irradiation, the electric field in the irradiated detector was higher than that in the pristine detector, resulting in a shorter width of the depletion region of the irradiated detector. Therefore, the irradiated detector showed an increased net current associated with photoconductive gain at bias voltages from 0 V to 200 V due to the high electric field. In contrast, the pristine detector showed transparent photoconductive gain only at higher bias voltages and dose rates, which could be attributed to the space charge effects [24].

**Figure 5:**
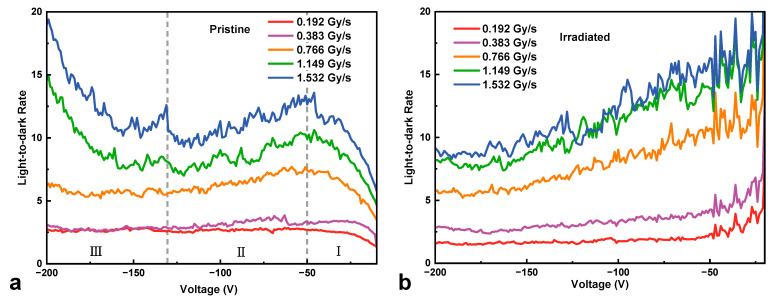
The light-to-dark ratio of (**a**) pristine and (**b**) irradiated HJD X-ray detectors at various dose rates against bias voltages.

Figure 5 shows the light-to-dark ratio of the HJD X-ray detector before and after irradiation as a function of the X-ray dose rate from 0.192 Gy∙s^−1^ to 1.532 Gy∙s^−1^. The irradiated HJD detector exhibited a higher light-to-dark ratio at low bias voltages while showing a lower light-to-dark ratio at high bias voltages than the pristine detector. Specifically, before irradiation, the pristine detector exhibited a non-monotonic light-to-dark ratio curve. In section Ⅰ, the net current increased with the width of the depletion region and faster than the low leakage current, and the light-to-dark ratio increased with the bias voltages. As the voltage increased, the rate of leakage current also rose, leading to a decrease in the light-to-dark ratio in section Ⅱ. When the bias voltage reached around −130 V, a high net current was obtained at high dose rates of 1.149 Gy∙s^−1^ and 1.532 Gy∙s^−1^, leading to an increased light-to-dark ratio with the bias voltages in section Ⅲ. In contrast, the irradiated HJD detector exhibited a decreased light-to-dark ratio overall. At low bias voltages (section I), the pristine detector showed a lower ratio than that of the irradiated detector because γ-ray irradiation caused an increase in the barrier height between p-NiO and n-Ga_2_O_3_, resulting in a decrease in leakage current. However, the net current of the irradiated device was restricted, and the leakage current continued to rise, leading to a steady decline in the light-to-dark ratio as the bias voltage increased.

To quantitatively evaluate the X-ray detection performances of the HJD X-ray detector before and after irradiation, the sensitivity (*S*) and the noise-equivalent dose rate (*NED*) were further calculated. The sensitivity is defined as the ratio between the net current and the incident X-ray dose rate reaching the detector (*D*) [23]:

(2) $$S=\frac{I_{net}}{D}$$

The noise-equivalent dose rate is defined as the incident X-ray dose rate that gives a signal-to-noise ratio of one in a 1 Hz bandwidth, which is a measure of the detection limitation of the detector and can be described by the following Equation (3) [23], where *I*_dark_ is the dark current (leakage current) of the detector, and *q* is the electron charge:

(3) $$NED=D\frac{\sqrt{2qI_{dark}}}{I_{net}}$$

**Figure 6:**
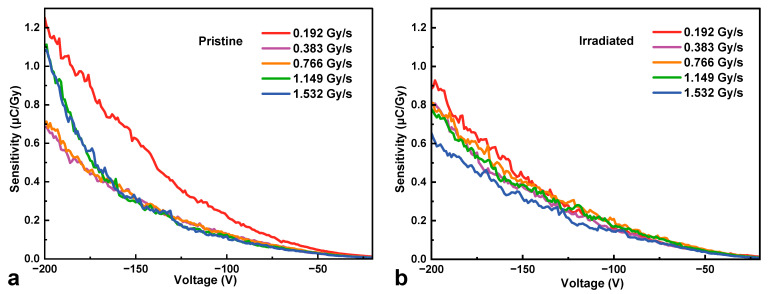
Sensitivity of (**a**) pristine and (**b**) irradiated HJD X-ray detectors at various dose rates against bias voltages.

**Figure 7:**
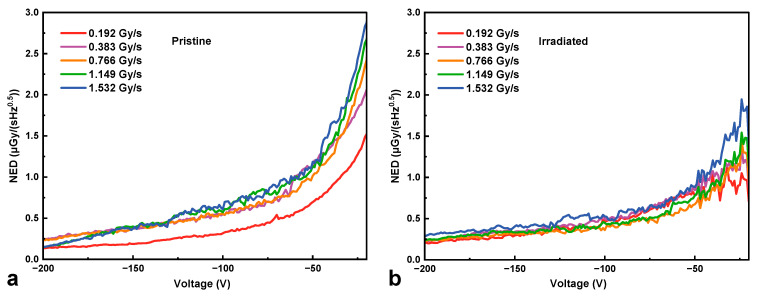
The noise equivalent dose rate (*NED*) of (**a**) pristine and (**b**) irradiated HJD X-ray detectors at various dose rates against bias voltages.

**Figure 8:**
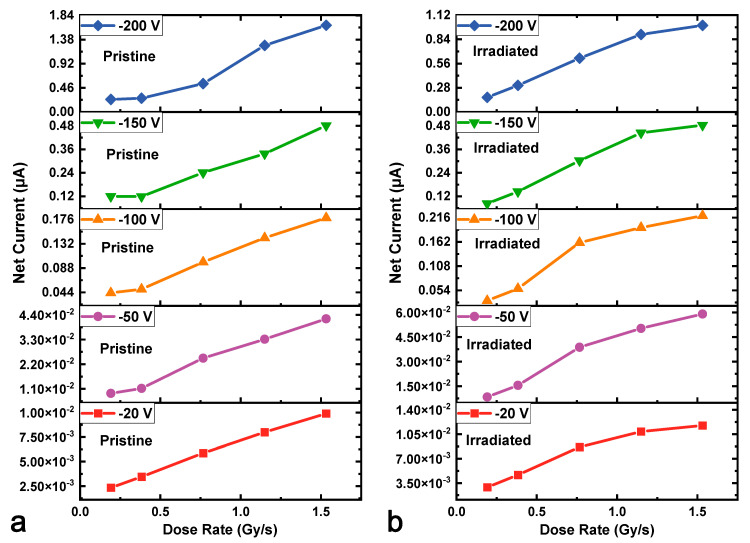
The net currents against X-ray dose rates of the (**a**) pristine and (**b**) irradiated HJD X-ray detectors at various bias voltages.

Figure 6 and Figure 7 plot the sensitivity and noise-equivalent dose rate versus reverse bias voltage at various dose rates from 0.192 Gy∙s^−1^ to 1.532 Gy∙s^−1^ for the HJD X-ray detectors before and after irradiation, respectively. The sensitivity of the HJD X-ray detector increased with the bias voltage before and after irradiation. Between the biased voltages from 0 to −160 V, the discrepancy of the sensitivity curve of the pristine detector at high dose rates was less than that of the net current, indicating a good linear relationship between net current and X-ray dose rates. The curves separated at bias voltages from −160 V to −200 V, and the output linearity was broken, which was associated with the trap-related photoconductive current and space charge effects. In contrast, for the HJD X-ray detector after irradiation, the sensitivity curves exhibited a clear difference, meaning that a sub-linear output occurred. The pristine HJD detectors exhibited linear outputs below −100 V, and the irradiated HJD detectors did not exhibit good linear outputs at various bias voltages. Hence, they are not suitable for X-ray dose rate detection. Moreover, attributed to the low dark current of the HJD X-ray detector, the *NED* decreased with bias voltages before and after irradiation. The *NED* of the irradiated HJD X-ray detector was higher than that before irradiation because the sensitivity decreased after the γ-ray irradiation. In addition, Figure 8 shows the net currents against X-ray dose rates of the pristine and irradiated HJD X-ray detectors at various bias voltages. The pristine detector exhibited sub-linearity only at a high bias voltage of −200 V. In comparison, the irradiated detector exhibited sub-linearity during the range from 0 V to −200 V, showing consistency with previous analysis.

**Figure 9:**
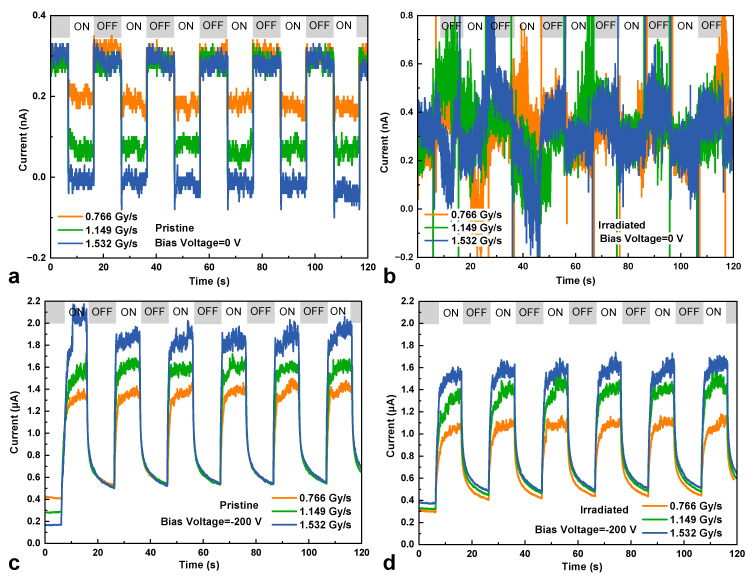
The transient response of the pristine and irradiated HJD X-ray detectors to the switching of X-ray illumination at biases of (**a**,**b**) 0 V and (**c**,**d**) −200 V.

Figure 9 reveals the transient response of the pristine and irradiated HJD X-ray detectors under switching X-ray illumination with a switching period of 10 s and a sampling rate of 20 ms. When the detector was biased at 0 V, the response current was equal to the photovoltaic current. In this mode, the response current was determined by the pn junction quality. It was clear that, after the γ-ray irradiation, the HJD X-ray detector exhibited a worse response to the switching X-ray at 0 V, meaning a worse junction characteristic of the HJD device caused by the irradiation. Moreover, The HJD before and after irradiation exhibited stable transient responses to the switching of X-ray illumination when biased at −200 V. Figure 9c,d show the absolute value of response current. However, the response time of the detector at −200 V was influenced by the photoconductive effect [7,19]. In this mode, the capture and release of the photo-generated carriers by the deep-level traps have a specific lifetime, leading to a relatively long response time. To study the trap-related time constant (*τ*) of the HJD X-ray detectors before and after irradiation, the fitting curve was performed at the dose rate of 0.766 Gy∙s^−1^ using exponential decay functions [25]:

(4) $$I\left(t\right)=I_{0}+\sum_{i=1}^{n}I_{i}\exp\left[-\left(t-t_{0}\right)/\tau_{i}\right]$$

where *I*(*t*) is the response current with a function of time of the HJD detector, *t* is the time, *t*_0_ is the initial time when the X-ray illumination is turned on or off, *I*_0_ is the steady current with/without X-ray illumination of the HJD detector, *τ* is the time constant, and *I* is the coefficient of time response for the *τ*.

**Figure 10:**
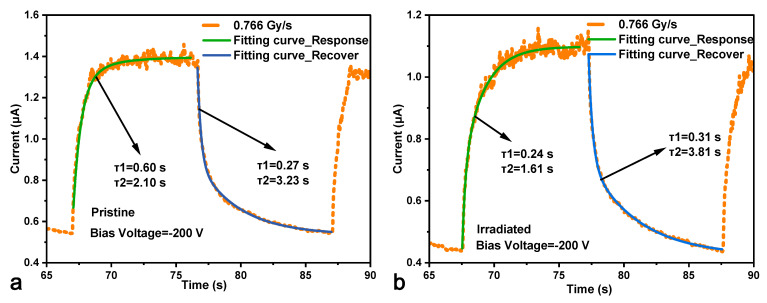
The transient response and the fitting curves of the (**a**) pristine and (**b**) irradiated HJD X-ray detectors to the switching of X-ray illumination at biases of −200 V in one period.

Figure 10 shows the fitting curves in one period of the HJD X-ray detector before and after irradiation. Two different time constants are obtained for both response curves to reveal the factors that affected the response/recovery time of the detector, where the response time is defined as the duration time required for the current to increase from 10% to 90% of the difference between the maximum and minimum currents. Conversely, the recovery current is defined as the time it takes for the current to decrease from 90% to 10% of that same difference. For the pristine detector, the response time constants were 0.60 s and 2.10 s with the coefficients 0.73 and 0.13, and the recovery time constants were 0.27 s and 3.23 s with the coefficients 0.99 and 0.37. For the irradiated detector, the response time constants were 0.24 s and 1.61 s with the coefficients 0.29 and 0.41, and the recovery time constants were 0.31 s and 3.81 s with the coefficients 0.55 and 0.32. More fitting information can be found in Table 1. Although the response time constants of the pristine detector seem longer than the irradiated detector, the coefficient of the short constant of the pristine detector was larger, leading to a faster response time. The longer response time indicated that the photoconductive mode was enhanced after the γ-ray irradiation. This result might be caused by the higher electric field in the irradiated detector due to the increased net carrier concentration or new type of traps generated by the γ-ray irradiation, which should be studied further.

**Figure 11:**
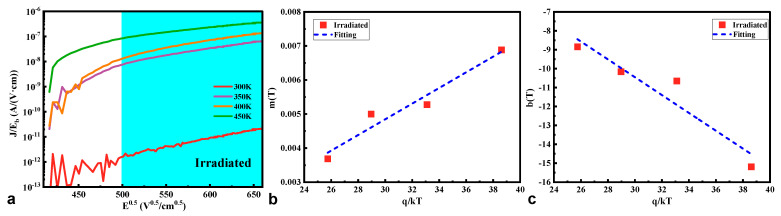
(**a**) The reverse *I*-*V*-*T* characteristics of the irradiated HJD X-ray detectors measured in a temperature range of 300 K to 450 K. (**b**) The *m*(T) curves and (**c**) the *b*(T) curves, as functions of q/kT for the measured irradiated HJD X-ray detectors (blue region).

In our previous study [7,23,25], the long-time constant-associated leakage current at high bias voltages mainly contributed to the electric-field-enhanced thermal emission process, such as the Poole–Frenkel emission (PFE). In this case, the reverse *I*-*V*-*T* characteristics of the HJD X-ray detector from 0 V to −200 V before and after irradiation were measured. The fitting results indicate that the PFE model was not suitable for the pristine detector but suitable for the irradiated detector. Figure 11a plots the *J*/*E* vs. *E*^0.5^ curve of the irradiated detector, according to the following Equation (5) [26]. To extract the relevant parameters in Equation (7) or (8) [15,26], *m*(*t*) and *b*(*t*) of the pristine and irradiated detectors are also plotted in Figure 11b,c. After the linear fitting process, the *ε*_∞_ and *qϕ_t_* were calculated, as shown in Table 2.

(5) $$J=CE_{b}\exp\left[-\frac{q\left(\phi_{t}-\sqrt{qE_{b}/\pi \varepsilon_{0}\varepsilon_{\infty }}\right)}{kT}\right]$$

(6) $$\log\left(J/E_{b}\right)\equiv m\left(T\right)\sqrt{E_{b}}+b\left(T\right)$$

(7) $$m\left(T\right)\equiv \frac{q}{2.3kT}\sqrt{\frac{q}{\pi \varepsilon_{0}\varepsilon_{\infty }}}$$

(8) $$b\left(T\right)\equiv -\frac{q\phi_{t}}{2.3kT}+\log C$$

where *E_b_* is the max electric field in the p-NiO/*β*-Ga_2_O_3_ HJD interface, q*ϕ*_t_ is the barrier height of electron emission from the trap states, *ε_0_* is the permittivity of vacuum, *ε*_∞_ is the high-frequency relative dielectric constant, *q* is the electron charge, *k* is the Boltzmann constant, *T* is the temperature, and *C* is a constant.

After the γ-ray irradiation, the *E*_b_ was increased due to the increased net carrier concentration of *β*-Ga_2_O_3_, and, thereby, the PFE process was enhanced. The Arrhenius plot of *m*(t) versus 1/kT allows us to estimate the high-frequency relative dielectric constant. The extracted *ε*_∞_ after irradiation was 2.27, close to the commonly used value of 3.60 [27], and the extracted energy level of the electron trap was located at 1.07 eV below the minimum value of the conduction band (*E*_c_) of *β*-Ga_2_O_3_ material. This trap was considered the primary trap state causing the PFE process [28] and was usually associated with donor-like oxygen divacancies in *β*-Ga_2_O_3_ [29].

## 4. Conclusions

In this study, a p-NiO/*β*-Ga_2_O_3_ HJD X-ray detector was tested under X-ray illumination before and after an online 13.5 kGy(Si) γ-ray irradiation at bias at −200 V. The X-ray response of the HJD X-ray detector was influenced by the trap-associated leakage current mechanism before and after irradiation. For instance, the net (response) current of the detector increased with bias voltages much faster than the expanding rate of the depletion region of HJD, and the output linearity against X-ray dose rates and response time characteristics worsened. Moreover, after irradiation, the net carrier concentration of *β*-Ga_2_O_3_ increased from 1.38 × 10^16^ cm^−3^ to 3.07 × 10^16^ cm^−3^, leading to a lower on-resistance, a larger barrier height of the HJD and a shorter width of the depletion region at a specific bias voltage. Thus, the reverse leakage current at low bias voltage was suppressed but increased at higher bias voltage due to the strengthening of the electric field. As a consequence, the PFE process was enhanced after the γ-ray irradiation. However, the underlying mechanisms for Ga_2_O_3_-based devices remain to be further investigated. In addition, the sensitivity of the HJD X-ray detector was lower at −200 V. The nonlinearity outputs against X-ray dose rates were observed from 0 V to −200 V. In addition, the transient response to switching X-ray illumination of the HJD X-ray detector was also degraded after irradiation; the response noise at 0 V increased, and the response time at −200 V increased from 2.92 s to 4.37 s. In short, the net carrier concentration of *β*-Ga_2_O_3_ increased, and the trap (*E*_c_—1.07 eV of *β*-Ga_2_O_3_ material)-associated PFE process enhanced after the γ-ray irradiation, limiting the sensitivity, linearity, and transient response performances of the HJD X-ray detector. In order to better improve the service life of the HJD X-ray detector in the irradiated environment, it is necessary to study the method of inhibiting the leakage current related to trap behavior.

## Figures and Tables

**Table 1:** The response/recovery time of the HJD detector before and after radiation.

Parameters	Pristine (Res./Rec.)	Irradiated (Res./Rec.)
Response time	2.92 s	4.37 s
Recovery time	5.03 s	5.16 s

**Table 2:** Extracted PFE parameters of the irradiated detector.

Parameters	Irradiated
*ε*_∞_	2.27
q*ϕ*_t_	1.07 eV

## Data Availability

The original contributions presented in this study are included in this article, and further inquiries can be directed to the corresponding author.
